# Involvement of ephrin receptor A4 in pancreatic cancer cell motility and invasion

**DOI:** 10.3892/ol.2014.2011

**Published:** 2014-03-28

**Authors:** CHENGLI LIU, HUI HUANG, CHENG WANG, YALIN KONG, HONGYI ZHANG

**Affiliations:** 1 Department of Hepatobiliary Surgery, Air Force General Hospital of PLA, Beijing 100142, P.R. China; 2 Department of Hepatobiliary Surgery, 309 Hospital of PLA, Beijing 100091, P.R. China

**Keywords:** ephrin receptor A4, invasion, motility, pancreatic cancer, matrix metalloproteinase-2, epithelial-mesenchymal transition

## Abstract

Ephrin (EPH) receptors can be classified into EPHA and EPHB receptors and are important in diverse cellular processes. EPHA4, a member of the EPHA receptors, has been demonstrated to be elevated in various human cancers and involved in the tumor progression. However, the role of EPHA4 in pancreatic cancer cells remains unclear. Therefore, the present study transfected Panc-1 and BxPC-3 cells with small interfering RNA (siRNA) to knockdown the expression of EPHA4. Wound healing and invasion assays were then performed to assess the effect of EPHA4 knockdown on the motility and invasion of pancreatic cancer cells. The results demonstrated that the knockdown of EPHA4 by siRNA inhibits the motility and invasion of pancreatic cancer cells. Furthermore, gelatin zymography assay showed that EPHA4 may regulate the activity of matrix metalloproteinase (MMP)-2. In addition, the knockdown of EPHA4 increased the expression of epithelial (E)-cadherin, as well as decreased the expression of Snail. Overall, these results suggested that EPHA4 may promote the motility and invasion of pancreatic cancer cells via the upregulation of MMP-2 and Snail, as well as the downregulation of E-cadherin. Thus, EPHA4 may act as a useful target for the treatment of pancreatic cancer.

## Introduction

Pancreatic cancer is a highly aggressive malignancy with a poor survival rate and invasion and metastasis are the most common cause of mortality in patients (1). Studies have demonstrated that tumor invasion and metastasis are complex processes that are regulated by various molecules. The majority of these molecules are ligands and receptors, which are involved in mediating the cell-to-cell or cell-to-extracellular matrix (ECM) interactions (2,3).

Ephrin (EPH) receptors belong to the family of receptor tyrosine kinases and have been demonstrated to be elevated in the majority of human cancers (4,5). EPH receptors can be classified into EPHA and EPHB receptors to which the EPHs are the ligands; divided into type A and B EPH ligands (6). Generally, type A EPH ligands are glycosylphosphatidylinisotol-anchored peripheral membrane molecules that bind EPHA receptors, while type B EPH ligands are transmembrane molecules that bind EPHB receptors. However, EPHA4 has the ability to bind to type A or B EPH ligands (7). Studies have shown that the expression level of EPHA4 is upregulated in gastric cancer (8). Furthermore, the overexpression of EPHA4 has been proven to correlate with liver metastasis in colorectal cancer (9). However, the role of EPHA4 in pancreatic cancer remains unclear. Therefore, the aim of the present study was to determine the effect of EPHA4 on the motility and invasion of pancreatic cancer cells.

## Materials and methods

### Antibodies

The antibodies against EPHA4 (mouse monoclonal antibody), epithelial (E)-cadherin (rabbit monoclonal antibody) and β-actin (mouse monoclonal antibody) were purchased from Santa Cruz Biotechnology, Inc. (Santa Cruz, CA, USA) and the antibody against Snail (rabbit monoclonal antibody) was obtained from Cell Signaling Technology, Inc. (Beverly, MA, USA).

### Cell lines and culture conditions

The pancreatic cancer cell lines, MIA PaCa-2, HAPC, SW1990, BxPC-3 and Panc-1, were purchased from the American Type Culture Collection (Manassas, VA, USA). All cells were incubated in Dulbecco’s modified Eagle’s medium (DMEM; Gibco-BRL, Gaithersburg, MD, USA) containing 10% fetal bovine serum (FBS; Hyclone, Logan, UT, USA) and cultured at 37°C in a humidified atmosphere of 5% CO_2_.

### Small interfering RNA (siRNA) and transfection

The following EPHA4 siRNA sequence was obtained from GenePharma (Shanghai, China): 5′-UCAUGAAGCUGAACACCGA-3′. As a control, the following scramble siRNA sequence was also used: 5′-UUCUCCGAACGUGUCACGU-3′. The cells were transfected with siRNAs using Lipofectamine reagent (Invitrogen Life Technologies, Carlsbad, CA, USA) according to the manufacturer’s instructions and further incubated for 48 h prior to being used in the subsequent experiments.

### Western blotting

The total protein of Panc-1 and BxPC-3 cells was extracted by radioimmunoprecipitation assay buffer with protease inhibitor and the concentration of the protein was examined using the Bicinchoninic acid (BCA) Assay kit (Applygen Technologies Inc., Beijing, China). The protein of each sample was then separated by SDS-PAGE (Invitrogen Life Technologies, Carlsbad, CA, USA) and transferred onto the polyvinylidene difluoride membranes (Invitrogen Life Technologies). The membranes were blocked with 5% non-fat milk for 1 h and incubated with the primary antibodies at 4°C overnight. Next, the membranes were probed with the secondary antibodies for 1 h at room temperature. Immunopositive bands were then detected and exposed to film following incubation with an enhanced chemiluminescence system (Applygen Technologies, Inc., Beijing, China). The expression of protein was normalized to the levels of β-actin.

### Wound healing assay

The cells were cultured in six-well plates at a density of 2×10^5^ cells/well and when the cells achieved 80% confluence, the cell monolayers were scratched with a sterile plastic pipette tip. The cells were then washed twice with phosphate-buffered saline and incubated with DMEM for 20 h. Images were captured at 0 and 20 h under an Olympus X71 inverted microscope (Olympus Corporation, Tokyo, Japan).

### Invasion assay

The invasion assay was performed using the 24-well Transwell inserts (Costar, Cambridge, MA, USA) and each filter of the Transwell was coated with Matrigel (BD Biosciences, Franklin Lakes, NJ, USA). The cells (1×10^5^ cells/well) were seeded onto the top chamber and 600 μl DMEM with 30% FBS was placed in the lower chamber. Following incubation for 20 h in a CO_2_ incubator, the invaded cells were fixed and stained with crystal violet (Amresco, Solon, OH, USA). Next, the invaded cells were observed under a microscope (YS100; Nikon, Tokyo, Japan) at a magnification of ×200. The mean number of cells in five random fields was calculated and the data are presented as a percentage of the invaded cells compared with the control.

### Gelatin zymography

The cells were transfected with siRNAs and incubated in a CO_2_ incubator for 48 h. The cell supernatant was then collected and concentrated at 8,000 × g for 30 min in a concentrator (Amicon Ultra concentrator, 30,000 Da MWCO; Millipore, Billerica, MA, USA). The concentration of the protein was determined by BCA assay and equal amounts of protein were separated on SDS-PAGE containing 1 mg/ml gelatin. The gel was washed with renaturing buffer (2.5% Triton X-100; Amresco) for 30 min at room temperature and then incubated with developing buffer [50 mM Tris-HCl buffer (pH 7.6), 5 mM CaCl_2_, 200 mM NaCl and 0.02% Brij-35; Invitrogen Life Technologies] overnight at 37°C. The gel was further stained with 0.5% Coomassie brilliant blue R-250 solution (Amresco) for 30 min, followed by destaining with 7.5% acetic acid solution containing 10% methanol. The areas of protease activity appeared as clear bands against the dark blue background.

### Statistical analysis

All experiments were repeated at least three times and the data are presented as the mean ± standard deviation. The results were analyzed using the SPSS 17.0 software (SPSS, Inc., Chicago, IL, USA) and the differences between the two groups were determined by Student’s t-test. P<0.05 was considered to indicate a statistically significant difference.

## Results

### Expression of EPHA4 in pancreatic cancer cells

**Figure 1:**
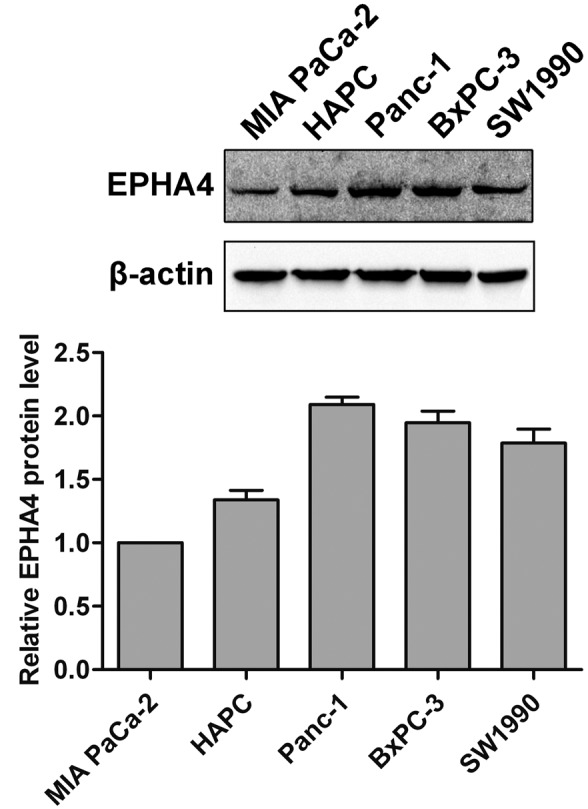
Protein level of EPHA4 observed in pancreatic cancer cells. Western blotting was performed to examine the expression of EPHA4 in MIA PaCa-2, HAPC, Panc-1, BxPC-3 and SW1990 cells. EPHA4, ephrin receptor A4.

The expression of EPHA4 in pancreatic cancer cell lines (MIA PaCa-2, HAPC, SW1990, BxPC-3 and Panc-1) was examined by western blotting. As shown in Fig. 1, EPHA4 was significantly expressed in all the examined pancreatic cancer cells.

### EPHA4 enhances the motility of pancreatic cancer cells

**Figure 2:**
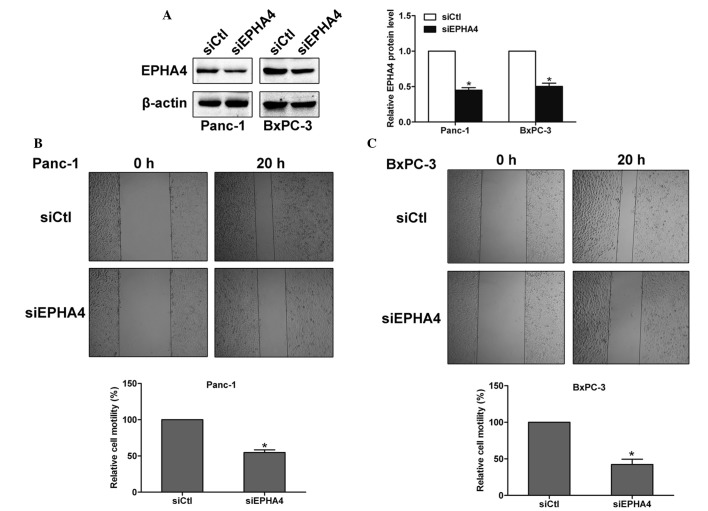
Knockdown of EPHA4 suppresses the motility of Panc-1 and BxPC-3 cells. (A) Knockdown efficiency was examined by western blotting following the transfection of Panc-1 and BxPC-3 cells with siEPHA4 or siCtl. Wound healing assay was performed to assess the effect of EPHA4 knockdown on the motility of (B) Panc-1 and (C) BxPC-3 cells. ^*^P<0.05, vs. siCtl. EPHA4, ephrin receptor A4; siRNA, small interfering RNA; siEPHA4, EPHA4 siRNA; siCtl, scramble siRNA.

To explore the function of EPHA4 in the pancreatic cancer cells, siRNA was introduced to silence the expression of EPHA4 in Panc-1 and BxPC-3 cells. The knockdown efficiency was examined by western blotting and the EPHA4 siRNA-transfected cells were shown to express lower levels of the EPHA4 protein (Fig. 2A). Next, a wound healing assay was performed in Panc-1 and BxPC-3 cells and the results showed that the motility of EPHA4 siRNA-transfected cells was significantly reduced compared with the control siRNA cells, which indicated that EPHA4 enhances the motility of pancreatic cancer cells (Fig. 2B and C).

### EPHA4 promotes the invasion of pancreatic cancer cells

**Figure 3:**
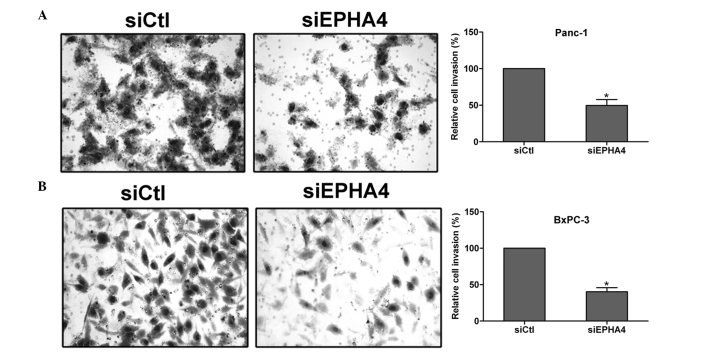
Knockdown of EPHA4 inhibits the invasion of pancreatic cancer cells. Following the knockdown of EPHA4, an invasion assay was performed on (A) Panc-1 and (B) BxPC-3 cells. The cells that had invaded through the Matrigel and filter were observed and counted under a microscope (magnification, ×200). ^*^P<0.05, vs. siCtl. EPHA4, ephrin receptor A4; siEPHA4, EPHA4 small interfering RNA; siCtl, scramble small interfering RNA.

Next, an invasion assay was performed to determine the function of EPHA4 in pancreatic cancer cell invasion. Notably, the knockdown of EPHA4 was found to suppress the invasion of Panc-1 and BxPC-3 cells, which suggested that EPHA4 is involved in the invasion of pancreatic cancer cells (Fig. 3A and B).

### EPHA4 is involved in the regulation of matrix metalloproteinase (MMP)-2 activity

**Figure 4:**
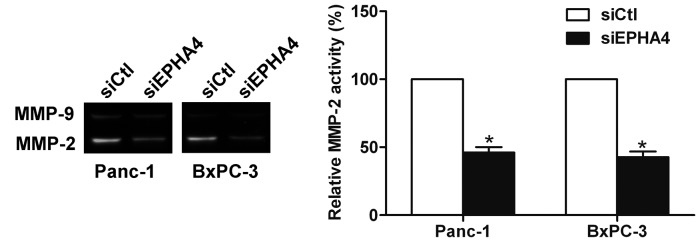
Knockdown of EPHA4 decreases the activity of MMP-2. Following transfection with siRNAs for 48 h, the cell supernatant was collected and the activity of MMP-2 in EPHA4 siRNA and control siRNA cells was detected by gelatin zymography. ^*^P<0.05, vs. siCtl. EPHA4, ephrin receptor A4; MMP, matrix metalloproteinase; siRNA, small interfering RNA; siEPHA4, EPHA4 siRNA; siCtl, scramble siRNA.

MMP-2 and MMP-9 are crucial in ECM degradation and are essential for the invasion and metastasis of pancreatic cancer (10). The knockdown of EPHA4 was found to inhibit the activity of MMP-2 by gelatin zymography, which suggested that EPHA4 is involved in the regulation of MMP-2 activity (Fig. 4).

### EPHA4 affects the expression of E-cadherin and Snail

**Figure 5:**
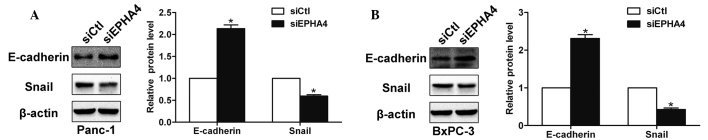
EPHA4 mediates the expression of E-cadherin and Snail. Following transfection with the siRNA for 48 h, the expression of E-cadherin and Snail in (A) Panc-1 and (B) BxPC-3 cells was examined by western blotting. ^*^P<0.05, vs. siCtl. EPHA4, ephrin receptor A4; E-cadherin, epithelial cadherin; siRNA, small interfering RNA; siEPHA4, EPHA4 siRNA; siCtl, scramble siRNA.

E-cadherin is an important cell adhesion molecule that is regulated by zinc-finger transcription factors, such as Snail, and is involved in epithelial-mesenchymal transition (EMT) and metastatic processes (11). Following the knockdown of EPHA4, the expression of E-cadherin was found to increase by western blotting, whereas the level of Snail was found to decrease (Fig. 5A and B). Thus, the results indicated that EPHA4 affects the expression of E-cadherin and Snail in pancreatic cancer cells.

## Discussion

As the largest family of receptor tyrosine kinases, the EPH receptors have been found to be overexpressed in a number of human cancers (12,13) and studies have shown that EPH receptors and their ligands are crucial in tumor progression (14,15). In addition, it has been reported that EPH receptors affect the cell-ECM attachment, thereby contributing to the invasion and metastasis of cancer (16). The current study demonstrated that EPHA4, a member of the EPH receptors, may promote the motility and invasion of pancreatic cancer cells. The knockdown of EPHA4 was found to suppress the activity of MMP-2, as well as increase the expression of E-cadherin and decrease the expression of Snail.

Upregulation of EPHA4 has been found in various types of tumors, such as gastric, colorectal and pancreatic cancer (8,9,17), and high expression of EPHA4 has been found to correlate with tumor progression, including the invasion, pathological stage and distant metastasis (18). Furthermore, overexpression of EPHA4 promotes the growth of pancreatic cancer cells (19) and enhances the proliferation and migration of glioma cells (20). The present study revealed that EPHA4 was greatly expressed in the pancreatic cancer cells. In addition, the knockdown of EPHA4 by siRNA inhibited the motility and invasion of pancreatic cancer cells, which indicated the involvement of EPHA4 in the motility and invasion of pancreatic cancer cells.

The MMPs are crucial enzymes for the degradation of the ECM and are involved in cancer cell invasion and metastasis (21). Furthermore, it has been reported that the overexpression of EPHA2 upregulates the expression of MMP-2 in Capan2 pancreatic ductal adenocarcinoma cells (22). The results of the present study demonstrated that EPHA4 has the ability to regulate the activity of MMP-2 in Panc-1 and BxPC-3 cells. As a cell adhesion molecule, E-cadherin can be negatively regulated by Snail and, therefore, acts as a crucial marker in EMT and the invasion of pancreatic cancer (23). Studies have found that EPHA4 mediates the EMT process of human hepatocellular carcinoma by downregulating the expression of E-cadherin and vimentin (24). The results of the current study also showed that the knockdown of EPHA4 may increase the expression of E-cadherin and decrease the expression of Snail in Panc-1 and BxPC-3 cells, which indicated that EPHA4 may be involved in the EMT process of pancreatic cancer cells.

In conclusion, the current study demonstrated that EPHA4 may promote the motility and invasion of pancreatic cancer cells. Furthermore, these processes may involve the upregulation of MMP-2 and Snail, as well as the downregulation of E-cadherin. However, further investigation is required to determine the signaling pathways by which EPHA4 enhances the motility and invasion of pancreatic cells.
